# Advances in methods for reducing mitochondrial DNA disease by replacing or manipulating the mitochondrial genome

**DOI:** 10.1042/EBC20170113

**Published:** 2018-06-27

**Authors:** Pavandeep K. Rai, Lyndsey Craven, Kurt Hoogewijs, Oliver M. Russell, Robert N. Lightowlers

**Affiliations:** 1 Wellcome Centre for Mitochondrial Research, Newcastle upon Tyne, U.K.; 2 Department of Organic and Macromolecular Chemistry, Ghent University, Krijgslaan 281 S4, 9000 Ghent, Belgium

**Keywords:** mitochondrial dysfunction, mtDNA, mitochondrial therapeuticsss

## Abstract

Mitochondrial DNA (mtDNA) is a multi-copy genome whose cell copy number varies depending on tissue type. Mutations in mtDNA can cause a wide spectrum of diseases. Mutated mtDNA is often found as a subset of the total mtDNA population in a cell or tissue, a situation known as heteroplasmy. As mitochondrial dysfunction only presents after a certain level of heteroplasmy has been acquired, ways to artificially reduce or replace the mutated species have been attempted. This review addresses recent approaches and advances in this field, focusing on the prevention of pathogenic mtDNA transfer via mitochondrial donation techniques such as maternal spindle transfer and pronuclear transfer in which mutated mtDNA in the oocyte or fertilized embryo is substituted with normal copies of the mitochondrial genome. This review also discusses the molecular targeting and cleavage of pathogenic mtDNA to shift heteroplasmy using antigenomic therapy and genome engineering techniques including Zinc-finger nucleases and transcription activator-like effector nucleases. Finally, it considers CRISPR technology and the unique difficulties that mitochondrial genome editing presents.

## Introduction

Mitochondria serve a vital role in normal cellular homoeostasis. A major function is the production of ATP via oxidative phosphorylation (OXPHOS) to drive cellular reactions. Mitochondria harbour multiple copies of their own circular genome. Mitochondrial DNA (mtDNA) encodes 22 tRNAs, 2 rRNAs and 13 polypeptides that comprise components of the electron transport chain and ATP synthase [1]. The maintenance of this genome is key for normal mitochondrial function but is susceptible to a high mutation rate, resulting in mtDNA mutations being established in the population at a greater rate than nuclear DNA [2]. The presence of mutated and wild-type (WT) mtDNA in the organelle is deemed heteroplasmy and is expressed as a percentage of the mutant genome present. Homoplasmy refers to the presence of only one population of mtDNA, either mutant or wild-type.

Mutations in mtDNA can cause mitochondrial disease. Mutation loads above a certain percentage can cause pathogenesis in a manner known as the threshold effect. Below this level, carriers can remain largely asymptomatic but those with heteroplasmy levels above this threshold can show severe symptoms. The threshold effect is ambiguous and is dependent upon several factors including the nature of the mtDNA mutation [3,4] or just the total amount of mtDNA [5]. The disease threshold can fall anywhere between 80% for disorders caused by point mutations, and 60% for those caused by single deletions [6,7].

The mitochondrial genome is maternally inherited. Inheritance of mtDNA is further complicated by the genetic bottleneck which occurs during embryonic development in female foetuses [8]. Mitochondrial DNA is segregated unevenly during primordial germline development and these segregated populations are replicated upon oocyte maturation, expanding the subset of mtDNA present within each cell. As a result, an asymptomatic mother could produce offspring with vastly varying levels of heteroplasmy [9,10]. The mechanisms of the genetic bottleneck are yet to be fully elucidated, making the prediction of the disease burden from one generation to another impossible to determine [11].

Current therapies for mitochondrial disease are limited to the alleviation of certain symptoms and curative potential is restricted to a few compounds currently in clinical trials, the outcomes of which are eagerly awaited [12]. Alternative approaches to eliminating mitochondrial disease involve the prevention of mtDNA transmission (mitochondrial donation) or the selective loss of mutated mtDNA (antigenomic methodology). Approaches to both these techniques vary widely and is the principle focus of this review. The merits and drawbacks of these methods are discussed below.

## Preventing mtDNA transmission

Currently, mothers diagnosed with mtDNA disease and wanting to conceive a child have several options available to prevent or reduce the likelihood of transmitting an mtDNA mutation. These options include adoption, use of a donor oocyte, prenatal diagnosis or preimplantation genetic diagnosis [13–15]. More recently, mitochondrial donation (MD) has become a credible alternative [16,17]. MD techniques include pronuclear transfer (PNT), maternal spindle transfer (MST), polar body transfer [18] and germinal vesicle transfer [16]. PNT and MST are the most extensively studied MD techniques and have been legally approved for use in the U.K. This review will discuss these two options further.

## Preimplantation genetic diagnosis

Preimplantation genetic diagnosis (PGD) is an IVF-based reproductive option that may be suitable for some women with mtDNA disease who wish to conceive a genetically related child with a reduced risk of severe disease [16]. The procedure involves the *in vitro* fertilization of oocytes harbouring pathogenic mtDNA mutations which are either cultured to the 6–8 cell stage before 1 or 2 cells are sampled for mutation load analysis [19] or cultured for 5 days and biopsied at the blastocyst stage [20]. PGD allows specialists to provide a better prognosis of disease risk but makes the assumption that the mutation load determined at the time of embryo biopsy is representative of the entire embryo and will remain stable during foetal development [21]. Although more research is required to determine exactly how mtDNA is segregated during embryonic development, data so far suggest that there is little variation in mutation load between different cells of preimplantation stage embryos and that children born following PGD harbour heteroplasmy levels similar to those reported at the embryonic stage [22]. Although effective in its ability to reduce the transmission of high levels of mutated mtDNA, PGD will not be suitable if the intending mother is homoplasmic or unable to produce embryos with low mutation levels [23]. Furthermore, identifying an embryo with an appropriate mutation load can be challenging, with acceptable mutation levels ranging from 5–30% depending on the specific mtDNA mutation and familial history [24]. This is subject to the threshold effect which can vary for different mutations [7]. The technique of PGD itself may also require a compromise between the identification of embryos with a low mutation load and those that are developmentally competent and likely to establish a pregnancy.

## Mitochondrial donation

Although PGD is an effective means of assuring the level of mutated mtDNA passed from mother to child is minimized, as mentioned above it will not be suitable for women with homoplasmic mtDNA mutations. This is because PGD will fail to identify an embryo with a low risk of mtDNA disease that could be selected for transfer. MD is a recent reproductive option that may be suitable for some patients with a high risk of having a child severely affected by mtDNA disease. The procedure can be performed at various stages of early development and involves removing the nuclear DNA from an oocyte or zygote containing mutated mtDNA and transferring this to an enucleated oocyte or zygote that contains WT mtDNA from a healthy donor.

## Maternal spindle transfer

**Figure 1:**
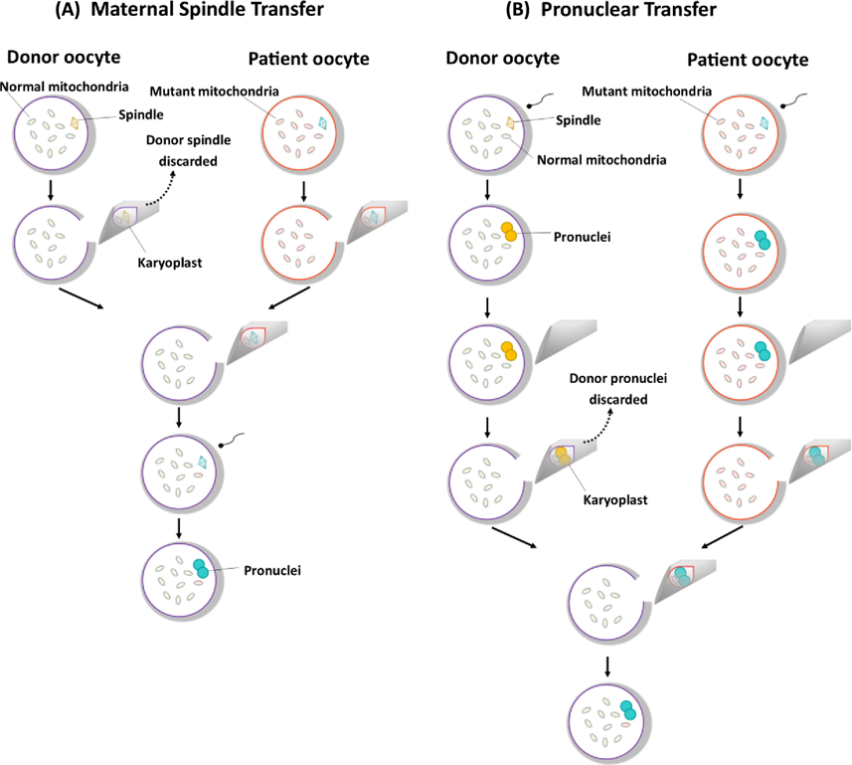
Mitochondrial donation (MD) techniques (**A**) Maternal spindle transfer (MST). (**B**) Pronuclear transfer (PNT). Both methods involve the removal of nuclear genetic material from patient and donor oocytes either pre- or post-fertilization. The nuclear genetic material from the patient oocyte or zygote is then transferred to the donor enucleated oocyte or zygote, resulting in a reconstructed oocyte or zygote that contains the nuclear genetic material from the intending parents and normal mitochondria from the donor.

The use of maternal spindle transfer (MST) to prevent transmission of mtDNA disease was first reported in [25]. In this study, rhesus macaque oocytes at the metaphase II stage of meiosis underwent spindle-chromosomal transfer to an enucleated donor oocyte (Figure 1A). Following this, the reconstructed oocytes were fertilized, cultured to the blastocyst stage and transferred to the uterus of female macaques. This resulted in the birth of three healthy offspring with undetectable levels of spindle-associated mtDNA in tested tissue and no health problems in follow-up studies [26]. The same group repeated the procedure in human oocytes but observed a higher rate of abnormal fertilization in zygotes that had undergone MST [27]. Despite this, embryos that fertilized normally following MST developed to the blastocyst stage at a comparable rate to unmanipulated controls. Human embryonic stem cells (hESCs) derived from MST blastocysts, however, revealed a reversal of mtDNA haplogroup from donor to maternal mtDNA in a limited number of hESC lines which initially contained low levels of maternal mtDNA carryover [27].

In 2017, the first live human birth was reported following the use of MST to reduce transmission of the m.8993T>G mutation causing Leigh syndrome. This resulted in an apparently healthy baby with <10% mutant mtDNA in tissues tested 2 days after the birth [28].

## Pronuclear transfer

Pronuclear transfer (PNT) was originally developed in 1983 [29] and involves removal of the pronuclei in a membrane-bound karyoplast from a fertilized zygote and transfer to an enucleated donor zygote (Figure 1B). The potential for this technique to prevent transmission of mtDNA disease was first demonstrated in mouse embryos carrying an mtDNA mutation [30], followed by a report using abnormally fertilized human zygotes [31]. In this proof of concept study, reconstructed embryos that developed following PNT had the capacity to reach the blastocyst stage. Optimization of the technique minimized the volume of cytoplasm removed with the pronuclei and reduced the mtDNA carryover to <2% [23], well below the threshold for mtDNA disease.

In 2016, the same group published the first preclinical evaluation of PNT using normally fertilized human embryos. Refinements to the PNT technique were required when using developmentally competent embryos, which included performing early PNT (ePNT) soon after meiosis II rather than close to the first mitotic division, resulting in an improved blastocyst development rate [32]. The study reported low levels of mtDNA carryover in PNT embryos but also observed a reversion to the maternal haplogroup in a limited number of hESC lines derived from PNT blastocysts. The study concluded that although carryover of mutant mtDNA could be greatly reduced, MD may not guarantee disease prevention.

In 2017, PNT was reported by popular media to have been performed at a fertility unit in Ukraine to overcome embryo arrest, resulting in the birth of an apparently healthy baby [33]. This clinical use of PNT has not been published, making it difficult to draw firm conclusions. The technique has been legally approved in the U.K. [17] but is only permitted to reduce the risk of serious mtDNA disease and is yet to be clinically applied.

Both MST and PNT harbour various advantages and disadvantages. MST is conducted on unfertilized oocytes, which may make this a preferred option for some. Alternatively, pronuclei are easier to visualize and contain the nuclear DNA surrounded by a membrane which may reduce the risk of potential damage [23]. The choice of technique used will depend on multiple factors including the expertise of the embryologist conducting the procedure and the legal stance of the country in which the procedure is to occur. Regardless of the technique used, long term follow-up of any child born from the procedure is important and should be performed to further assess the safety of the technique.

An important consideration for any novel reproductive technology is ensuring an adequate level of safety and efficacy has been demonstrated before clinical application. The reversion of donor mtDNA to the maternal mtDNA haplogroup observed in some hESC lines is a concern but given that hESC development varies greatly from foetal development, the clinical implication of this observation is unclear [34]. Several safeguards have been proposed to minimize the risk, such as haplogroup matching the patient and donor mtDNA. Another recommendation is that MD should be combined with prenatal testing to reduce the risk of having a child severely affected by mtDNA disease [32].

## Approaches to shifting mtDNA heteroplasmy

An alternative approach to the treatment of disease caused by mtDNA is to target the mitochondrial genome itself. The threshold effect highlights the fact that if the ratio of WT to mutant mtDNA can be adjusted, the presence of WT molecules will overcome the effect of variant ones and hence correct the defect [35]. The direct manipulation of the heteroplasmy level or the supplementation of an existing wild-type population to reduce heteroplasmy would therefore alleviate symptoms without the requirement for total removal of the mutant genome [36]. Several approaches have been trialled in this approach, with the most advanced outlined below.

## Antigenomic mtDNA therapy

**Figure 2:**
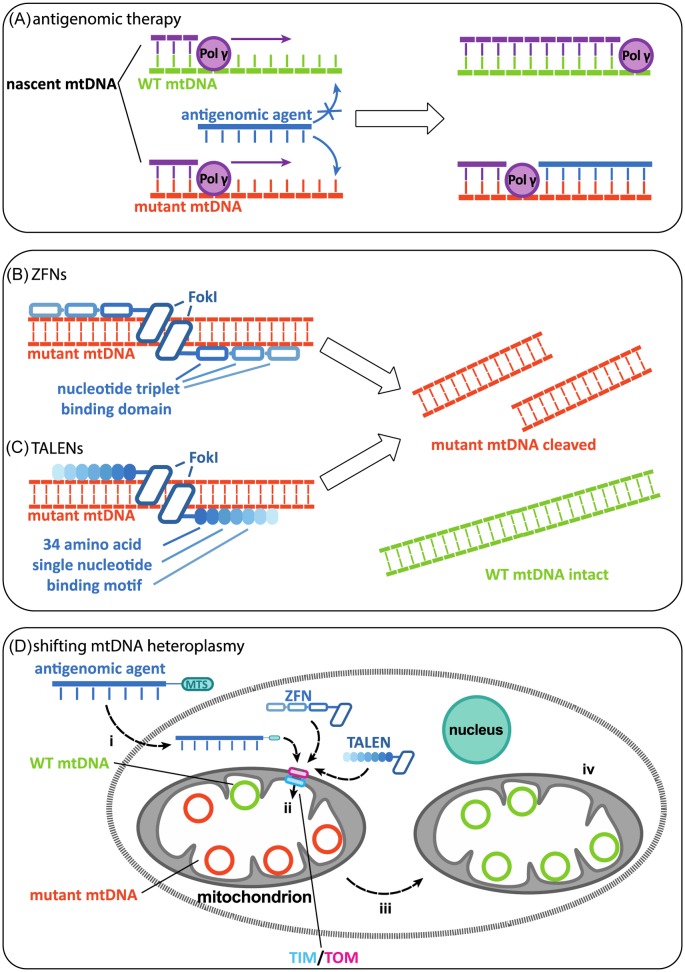
Approaches to shifting heteroplasmy (**A**) Reduction in the mutant mtDNA levels by selectively inhibiting the Pol γ replication of mutant mtDNA by a short complementary oligonucleotide. (**B**) Binding and cleavage of mutant mtDNA by ZFNs or (**C**) TALENs. (**D**) Overall summary of the approaches: (i) delivery of an antigenomic agent into the cell, (ii) translocation of an antigenomic agent/ZFN/TALEN into the mitochondrial matrix, and (iii and iv) the treatment should result in mitochondria containing (almost) exclusively WT mtDNA.

One possible approach is to reverse disease progression by selectively inhibiting the replication of the mutant mtDNA, allowing the WT mtDNA population to propagate (Figure 2). Indeed, selective inhibition was successfully demonstrated in an *in vitro* replication run-off experiment using peptide nucleic acids (PNA) complementary to mutant mtDNA [37]. Delivery of these complex polymers (11- to 14-mers) to the mitochondrial matrix is challenging, but appeared to be possible by fusing the PNA with either a triphenyl-phosphonium (TPP) moiety or the COX VIII mitochondria targeting sequence (MTS) [38,39]. Unfortunately, the early *in vitro* success could not be expanded to cultured heteroplasmic cells [40]. A possible explanation for this was the inability of the PNA to bind the mtDNA, possibly due to competition with the more abundant RNA, or its rapid removal during replication [41]. In addition, doubts arose concerning the reliability of current methods to confirm and detect uptake into the mitochondrial matrix [42]. To circumvent these issues, a new methodology named “ClickIn” was developed [43,44]. In this method, MitoOct, a bio-orthogonally reactive TPP accumulates in the mitochondrial matrix of energized mitochondria, where it will react at an accelerated rate with azide labelled PNA, therefore selectively labelling matrix localized PNA. Using this strategy, the authors were able to show that a tetrameric PNA molecule conjugated to the COX VIII MTS could be translocated, though at a cost in efficiency compared with the MTS without PNA attached. Future experiments will determine whether experiments can be repeated for longer, therapeutically relevant PNAs, or if oligonucleotides with different backbone chemistries should be explored.

Research into an antigenomic strategy is not restricted to PNA. In recent years, researchers have been exploring the potential of RNA as an antigenomic agent for mtDNA diseases. Controversy exists concerning the veracity of import and method of RNA uptake into mitochondria [45]. However, it has been reported that expression of a putative mitochondrially targeted antigenomic RNA in cultured cells led to a shift in heteroplasmy [46]. Such a report is exciting, but until the molecular mechanisms of RNA transport across the inner mitochondrial membrane can be fully resolved, the question of whether RNA is naturally taken up into the mitochondrial matrix is likely to remain contested.

## Targeted restriction endonucleases

An alternative method to alter mtDNA heteroplasmy is the utilization of restriction endonucleases. Restriction endonucleases (REs) can be targeted to the mitochondrion and produce a double strand break at a site specific region present in only mutant mtDNA molecules [47,48].

In 2001, Srivastava and Moraes [47] demonstrated the use of REs in the manipulation of mtDNA heteroplasmy by employing *Pst*I in a mouse-rat cybrid cell line in which only mouse mtDNA contained two *Pst*l sites. This experiment demonstrated a significant shift in mtDNA heteroplasmy. Simultaneous work by Tanaka et al. [48] demonstrated the potential for mitochondrially targeted *Sma*l to alter heteroplasmy in cybrid human cell lines harbouring the m.8993T>G mutation in *MT-ATP6*. The group achieved total elimination of variant mtDNA after repeated transfection cycles. The presence of the variant produces a *Sma*I restriction site which was also targeted by Alexeyev et al. [49] who used adenoviral delivery of the *Sma*I isoschizomer *Xma*I (accompanied by a *Sma*I methytransferase) to cybrid lines, causing a substantial shift in heteroplasmy.

In 2005, Bayona-Bafaluy et al. [50] reported a restriction endonuclease strategy in which mitochondria from mice heteroplasmic for mtDNA from BALB/c and NZB strains were targeted by mitochondrially targeted restriction endonuclease *Apa*LI (mito-*Apa*LI). The presence of an *Apa*LI restriction site in BALB/c but not NZB mtDNA caused transient depletion of the mtDNA copy number followed by amplification of the remaining mtDNA, resulting in a shift towards the NZB haplogroup. The group further developed this model by using an adenoviral construct to deliver mito-*Sca*I to mitochondria, targeting three restriction sites on BALB/c mtDNA and five on NZB [51]. The same adenoviral vector was delivered to the liver and skeletal muscle of the heteroplasmic mice and caused a shift in heteroplasmy towards BALB/c predominance but was not as efficient as single cleavage by mito-*Apa*LI. Mito-*Apa*LI has also been delivered to liver, cardiac and skeletal muscle [52,53] by exploiting advances in AAV6, Ad5 and AAV9 vector optimization [54–56]. Finally, mito-*Apa*L1 has also been used to reduce the levels of heteroplasmy in single cell murine embryos ([57], see below).

Although several promising studies have been conducted, the use of available REs relies on the creation of unique restriction sites via pathogenic variants on mutant mtDNA and harbours the potential for off-target effects on the nuclear genome [58]. One approach for overcoming this limitation is the use of engineered endonucleases to target specific sequences on the mitochondrial genome that cannot be targeted by naturally occurring REs.

## Zinc-finger nucleases

Cys2–His2 Zinc-finger nucleases (ZFNs) are short ∼30 amino acid sequences folded into an antiparallel β sheet, and α helix motif containing a single zinc ion [59] These finger like DNA-binding domains can be attached to the endonuclease *Fok*I at the C-terminal, which, upon dimerization, introduces a double strand break in target DNA sequences [60,61]. Historically, two ZFN monomers are targeted to adjacent sites on a complementary DNA strand allowing *Fok*I dimerization. In 2006, Minczuk et al. [62] engineered zinc finger proteins (ZFPs) with an MTS alongside a nuclear export sequence. In this proof of principle study, ZFPs targeting the m.8993T>G mutation were combined with the catalytic domain of the human DNMT3a DNA methyltransferase. Methylation of 5C cytosines in CpG sites near the target sequence was observed in 23% of mutant clones.

A further development in the use of ZFNs was the creation of a monomer with dual F*ok*l domains separated by a 35-residue linker able to bind to complementary DNA strands [63]. This quasi-dimer was able to access the mitochondrial matrix and show mtDNA binding selectivity. The ZFN was used to selectively cleave the m.8993T>G variant mtDNA in heteroplasmic cybrid cell lines, resulting in an increase in WT mtDNA load. However, long-term continuous expression of ZFNs was found to reduce clone survival suggesting possible toxic effects and a loss of total mtDNA copy number [63].

More recently, Gammage et al. produced mitochondrial ZFNs (mtZFNs) to target the m.8993T>G point mutation and the 4977 bp common deletion. The group improved on their previous design by developing individual ZF monomers to target complementary DNA strands. Modified *Fok*I nucleases were employed that only cleave as a heterodimer. Cybrid cell lines harbouring the m.8993T>G mutation were transfected for 18 days and displayed a 7–17% increase in WT mtDNA. Furthermore, the common deletion was targeted by the creation of a range of monomers to target the deletion breakpoints, thus only allowing F*ok*I dimerization and cleavage when the breakpoints are adjacent to one another as in the deleted species, rather than far apart as in the WT mtDNA molecule. Southern blotting showed an increase in WT mtDNA from 15 to ∼76% in HOS H39 cells. Functional analysis of these cells demonstrated an increase in oxidative capacity [64].

This work has shown great promise for the use of ZFNs in the elimination of mtDNA mutations. Although genetic engineering has improved the efficacy of the proteins, off-target cleavage in the nucleus by more standard ZFN dimers has been reported. Further, it is difficult to rule out the possibility of low level mis-localization of mtZFNs to the nucleus [62,64]. To address the formal possibility of off-target effects for mitochondrially targeted ZFNs, Gammage et al. [65] were able to show that such effects could be minimized by the careful control of the levels of expression of the ZFNs.

## Transcription activator-like effector nucleases

An alternative to the use of ZFNs are transcription activator-like effector nucleases (TALENs). These comprise 34 amino acid motifs that each binds one base pair in a DNA sequence. The 12th and 13th amino acids are key in determining base recognition and are known as the variable di-residues or RVDs (for review see [66]). TALENs also contain *Fok*I restriction endonuclease activity that initiates double strand cleavage upon dimerization after the site specific binding of two TALEN monomers on complementary DNA strands. Mitochondrially targeted TALENs (mitoTALENs) have been successfully employed by Bacman et al. [67] in cybrid cells containing the common deletion (m.8483–13459) and the m.14459G>A point mutation underlying Leber’s hereditary optic neuropathy and dystonia. The group selectively cut deleted DNA by targeting mitoTALENs to the breakpoint sequences, which would only be in close enough proximity to dimerize when bound to mutant mtDNA. To allow site-specific cleavage, a number of repeats [10–15] can be employed to produce each TALEN monomer. The group then continued their work by designing more efficient mitoTALENs [68]. Specifically, they utilized shorter repeat sequences and optimized mitochondrial targeting by comparing efficiencies of various MTSs. The group used the newly designed mitoTALENs to shift the heteroplasmy levels of cybrid cells harbouring the m.8344A>G mutation associated with myoclonic epilepsy and ragged red fibres and the m.13513G>A mutation associated with mitochondrial encephalomyopathy, lactic acidosis and stroke-like episodes or Leigh syndrome. The shorter mitoTALENs were still able to exert a shift in heteroplasmy with a resultant improvement of mitochondrial OXPHOS. This study is important in the pioneering work of mitoTALEN development, as a major disadvantage in their use has been the large size of the constructs needed to express such large molecules, which has particularly made the packaging and delivery of TALENs in adenoviral vectors, challenging [68].

The use of mitoTALENs has also been considered as a method for helping to prevent the transmission of pathogenic mtDNA in man [57]. Human oocytes heteroplasmic for the pathogenic m.14459G>A or the m.9176T>C were prepared and mRNA expressing mitoTALENs specific to each mutation were injected into the respective oocytes. After 48 h, analysis of mtDNA confirmed a significant decrease in both of the mutant populations. Total copy number was also noted, consistent with the absence of mtDNA replication in mature oocytes.

## CRISPR technology

Many permutations of the CRISPR–Cas9 genome editing system have been employed in the past few years and there has been great interest in its use to repair pathogenic nuclear mutations. Targeting of the gene editing system is mediated via a guide RNA. Therefore, to use the CRISPR–Cas9 system to manipulate the mitochondrial genome, gRNA would need to be imported. As intimated above, there is conflicting opinion as to whether RNA is imported into mammalian mitochondria. The absence of an RNA component in mitochondrial RNase P, an enzyme that usually has an RNA component, taken together with the observation that the mitoribosome employs a structural RNA that is a mitochondrially encoded tRNA and not a more standard 5S RNA component has led many to question whether mammalian mitochondria need to import RNA. This has to be considered against a plethora of publications reporting mitochondrial import and function both of exogenous RNA [69] or indeed of miRNA [70]. Strikingly, in 2015, Jo et al. [71] published a report claiming the use of CRISPR–Cas9 to shift mtDNA heteroplasmy in mammalian cells. The group designed sgRNA against *MT-CO1* or *MT-CO3* and expressed a modified Cas9 protein with a MTS. After 5 days of expression, a decrease in copy number and cellular proliferation was observed. However these data are controversial [45] and to our knowledge, no other apparently successful use of CRISPR–Cas9 for editing mtDNA has been published. Being able to edit the mitochondrial genome at will is a prime objective for many in the field of mammalian mitochondrial genetics and therapeutics. Clearly, if mitochondrial RNA import is a reality we would expect to see many future publications using such an approach.

## Conclusion

The use of PGD has greatly improved the likelihood of women with high levels of pathogenic mtDNA mutations to have children with a reduced risk of disease, but for a subset of patients for whom PGD is likely to fail, MD poses an appealing alternative. Technical and ethical differences between MD techniques may influence the decision to use one method over another but at present, there is no evidence to suggest that either MST or PNT is preferable. The important influence of heteroplasmy on threshold effects in relation to pathogenic mtDNA mutations has made the development of technologies to either prevent or reduce mtDNA transmission or to shift heteroplasmy, attractive therapeutic targets. A new method to quantify mitochondrial import of antigenomic agents has rekindled interests in preventing the replication of mutant mtDNA as a therapeutic method for mtDNA disease. Finally, methods that cause changes in mtDNA heteroplasmy have also shown great success *in vitro* and some advances *in vivo*. Both mtZFNs and mitoTALENs have been shown to eliminate specific mtDNA molecules. Further development of these techniques could allow their use as gene therapies in the clinical future.

## Summary

- Mutations in mitochondrial DNA can lead to the development of mitochondrial disease.
- Methods for avoiding clinical manifestation of these diseases include preventing the transmission of mutated mtDNA, either by selecting embryos with low levels of mutant mtDNA using PGD or via the use of mitochondrial donation, which aims to reduce or eliminate mutant mtDNA.
- In the cases where mtDNA disease manifests after birth, mtDNA gene therapies are being pioneered to reduce the level of mutant mtDNA and increase the ratio of WT mtDNA present.
- Gene editing methods that have been pioneered include the use of antigenomic therapies, ZFNs and TALENs.

## Abbreviations

MD: mitochondrial donation
mito-*Apa*LI: mitochondrially targeted restriction endonuclease *Apa*LI
mitoTALEN: mitochondrially targeted TALEN
MST: maternal spindle transfer
mtDNA: mitochondrial DNA
MTS: mitochondria targeting sequence
mtZFN: mitochondrially targeted Zinc-finger nuclease
PGD: prenatal genetic diagnosis
PNA: peptide nucleic acid
PNT: pronuclear transfer
TALEN: transcription activator-like effector nuclease
TPP: triphenyl-phosphonium
ZFN: Zinc-finger nuclease
